# Comparison between thulium laser resection of prostate and transurethral plasmakinetic resection of prostate or transurethral resection of prostate

**DOI:** 10.1038/srep14542

**Published:** 2015-10-07

**Authors:** Hong DeCao, Jia Wang, Yu Huang, Ren LiangLiu, Hao JunLei, Liang Gao, Zhuang Tang, Chun YingHu, Xiang Li, Hong JiuYuan, Qiang Dong, Qiang Wei

**Affiliations:** 1 Department of Urology, West China Hospital of Sichuan University, Chengdu, China; 2 Department of Geriatrics, West China Hospital of Sichuan University, Chengdu, China

## Abstract

Benign prostatic hyperplasia (BPH) is one of the most common diseases in middle-aged and elderly men. In the present study, we aimed to compare the efficacy and safety of thulium laser resection of the prostate (TMLRP) with either transurethral plasmakinetic resection of the prostate (TUPKP) or transurethral resection of the prostate (TURP). A literature search was performed, eventually, 14 studies involving 1587 patients were included. Forest plots were produced by using Revman 5.2.0 software. Our meta-analysis showed that operation time, decrease in hemoglobin level, length of hospital stay, catheterization time, and development of urethral stricture significantly differed, whereas the transitory urge incontinence rate, urinary tract infection rate, and recatheterization rate did not significantly differ between TMLRP and either TURP or TUPKP. The blood transfusion rate was significantly different between TMLRP and TURP, but not between TMLRP and TUPKP. In addition, the retrograde ejaculation rate between TMLRP and TURP did not significantly differ. At 1, 3, 6, and 12 months of postoperative follow-up, the maximum flow rate, post**-**void residual, quality of life, and International Prostate Symptom Score did not significantly differ among the procedures. Thus, the findings of this study indicate that TMLRP may be a safe and feasible alternative.

Benign prostatic hyperplasia (BPH) is one of the most common diseases in middle-aged and elderly men, and is one of the most common causes of lower urinary tract symptoms (LUTS)^1^. LUTS cause discomfort, and often have a significant impact on the quality of life (QoL) of patients. Approximately 50–60% of men aged >60 years suffer from BPH and the associated LUTS^2^,^3^. Surgical removal is an appropriate treatment option for patients with moderate to severe LUTS. Although traditional transurethral resection of the prostate (TURP) has been considered as the standard surgical procedure for patients with BPH for decades^4^,^5^, it has been associated with the development of significant complications^6^,^7^.

With the development of new scientific and technological methods, more advanced surgical techniques are now being employed in BPH treatment. Transurethral plasmakinetic prostatectomy (TUPKP) is one such procedure; it can be performed with normal saline (NaCl 0.9%) irrigation and overcomes a fundamental disadvantage of TURP, which ensures that surgeons have more time to safely resect larger prostates^1^,^8^.

The thulium laser resection of the prostate (TMLRP) technique is also a relatively new approach, and was first reported in 2005^1^. In TMLRP, a wavelength of approximately 2 μm is emitted in continuous-wave mode, thus enabling the precise incision of tissue by using a wavelength that matches the water absorption peak of 1.92 μm in tissue. Thus, the procedure ensures more effective resection and vaporization of prostate tissue^8^. In addition, because TMLRP achieves excellent urine clarity after surgery, patients do not require bladder irrigation. Furthermore, the risk of TUR syndrome is decreased because TMLRP involves the use of physiologic saline as the irrigation fluid^8^,^9^.

Several clinical trials have proven that the aforementioned techniques are all safe and effective for patients with BPH^2^,^3^,^4^,^8^,^10^. However, no published multinational study or other evidence definitively declares the superiority of the TMLRP technique over the others. Therefore, we performed this systematic review and meta-analysis to assess the TMLRP technique in comparison with either TURP or TUPKP.

## Results

### Literature search

**Figure 1:**
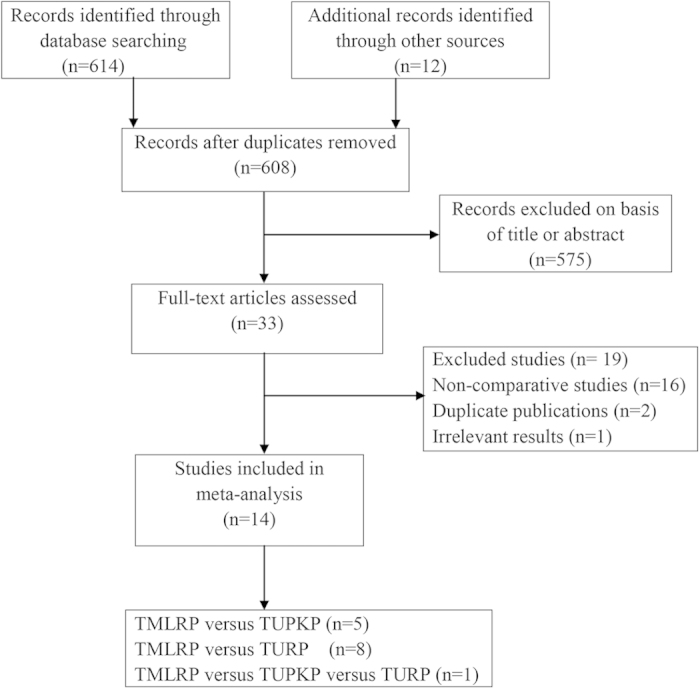
Flow diagram of evidence acquisition.

We formulated an exhaustive search strategy—using a combination of electronic database and manual searches—to identify all relevant studies. Our search yielded 614 studies, of which 576 were excluded due to irrelevance, based on their titles and abstracts. After a quality assessment, we finally included 14 studies^11^,^12^,^13^,^14^,^15^,^16^,^17^,^18^,^19^,^20^,^21^,^22^,^23^,^24^ in this meta-analysis. Our literature screening process is summarized in Fig. 1.

### Characteristics of the included studies

Five studies comparing TMLRP to TUPKP^11^,^12^,^13^,^14^,^15^; one study comparing TMLRP, TURP, and TUPKP^16^; and eight studies comparing TMLRP to TURP^17^,^18^,^19^,^20^,^21^,^22^,^23^,^24^ were included. Thus, a total of 1587 patients were included in this study from the fourteen trials. Among these patients, 789 underwent TMLRP, 438 underwent TURP, and 360 underwent TUPKP. Ten studies were randomized controlled trials (RCTs)^12^,^13^,^14^,^17^,^18^,^19^,^20^,^21^,^22^,^24^ and four were clinical controlled trials (CCTs)^11^,^15^,^16^,^23^. All included studies reported the number of participating patients, their age, follow-up time, maximum flow rate (Qmax), and International Prostate Symptom Score (IPSS). Ten studies reported prostate volume^12^,^13^,^14^,^15^,^17^,^18^,^19^,^20^,^21^,^22^,^23^,^24^, three studies failed to report prostate specific antigen (PSA) values^16^,^20^,^23^, one study failed to report post**-**void residual (PVR)^23^, and two studies failed to report QoL scores^11^,^17^. The basic characteristics and quality assessments of the included studies are summarized in Table 1.

### Meta-analysis results

#### Efficacy

##### Qmax

No significant difference was noted in Qmax between TMLRP and either TURP or TUPKP during the 1-, 3-, 6-, and 12-month postoperative follow-up (all *p* > 0.05, Table 2).

##### PVR

Pooled data revealed that there were no significant differences between TMLRP and either TURP or TMLRP in terms of the PVR during the 1-, 3-, 6-, and 12-month postoperative follow-up (all *p* ≥ 0.05, Table 2).

##### QoL

TMLRP was associated with a higher QoL score than TURP or TUPKP at the 1-month postoperative follow-up (*p* < 0.0001 and *p *= 0.003, respectively), although there were no significant differences during the 3-, 6-, and 12-month postoperative follow-up (*p* > 0.05, Table 2).

##### IPSS

TMLRP was associated with an IPSS similar to that of TURP or TUPKP at the 1-, 3-, 6-, and 12-month postoperative follow-up (all *p* > 0.05, Table 2).

#### Safety

##### Operative time (minutes)

**Figure 2:**
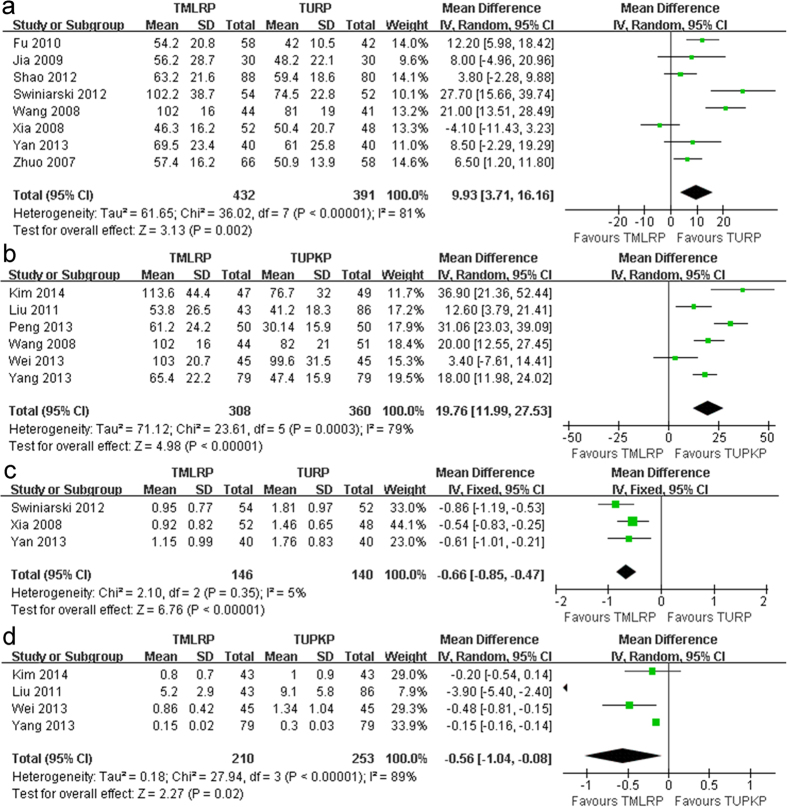
(**a**) Pooled estimate of operative time between TMLRP and TURP. (**b**) Pooled estimate of operative time between TMLRP and TUPKP. (**c**) Pooled estimate of hemoglobin decreased between TMLRP and TURP. (**d**) Pooled estimate of hemoglobin decreased between TMLRP and TUPKP.

The operative time was recorded in 13 studies^11^,^12^,^13^,^14^,^15^,^16^,^17^,^19^,^20^,^21^,^22^,^23^,^24^ that included 1491 patients. Analysis of these studies indicated that the operative time for the TMLRP group was significantly longer than that for the TURP group (mean difference [MD]: 9.93; 95% confidence interval [CI]: 3.71 to 16.16; *p* = 0.002; I^2^: 81%; Fig. 2a). Moreover, our meta-analysis showed that the operative time for the TMLRP group was longer than that for the TUPKP group (MD: 19.76; 95% CI: 11.99 to 27.53; *p* < 0.001; I^2^: 79%; Fig. 2b). This meta-analysis demonstrated that TMLRP involved a longer operation time than either of the other techniques.

##### Hemoglobin level decrease (g/dL)

We extracted data on the decrease in hemoglobin levels from seven relevant studies^11^,^12^,^13^,^15^,^17^,^19^,^20^. The decrease in hemoglobin level was significantly lower for the TMLRP group than for the TURP group (MD: −0.66; 95% CI: −0.85 to −0.47; *p* < 0.001; I^2^: 5%; Fig. 2c) or TUPKP group (MD: −0.56; 95% CI: −1.04 to −0.08; *p* = 0.02; I^2^: 89%; Fig. 2d).

##### Length of hospital stay (days)

**Figure 3:**
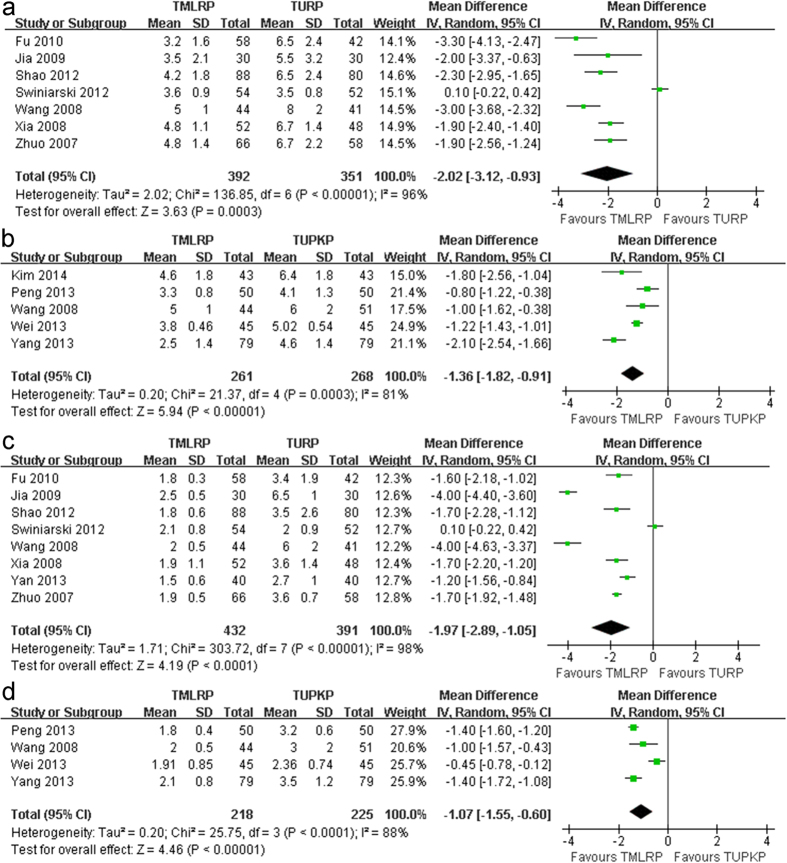
(**a**) Pooled estimate of hospital stay between TMLRP and TURP. (**b**) Pooled estimate of hospital stay between TMLRP and TUPKP. (**c**) Pooled estimate of catheterization time between TMLRP and TURP. (**d**) Pooled estimate of catheterization time between TMLRP and TURP.

This outcome was reported in 11 relevant studies^11^,^12^,^13^,^14^,^16^,^19^,^20^,^21^,^22^,^23^,^24^. The pooled analysis indicated that the TMLRP group had a shorter length of hospital stay than either the TURP group (MD: −2.02; 95% CI: −3.12 to −0.93; *p* < 0.001; I^2^: 96%; Fig. 3a) or the TUPKP group (MD: −1.36; 95% CI: −1.82 to −0.91; *p *< 0.001; I^2^: 81%; Fig. 3b).

##### Catheterization time (days)

Eleven relevant studies^12^,^13^,^14^,^16^,^17^,^19^,^20^,^21^,^22^,^23^,^24^ including 1266 patients reported on the catheterization time. The pooled data demonstrated a markedly shorter catheterization time for the TMLRP group as compared to either the TURP group (MD: −1.97; 95% CI: −2.89 to −1.05; *p* < 0.001; I^2^: 98%; Fig. 3c) or the TUPKP group (MD: −1.07; 95% CI: −1.55 to −0.60; *p *< 0.001; I^2^: 88%; Fig. 3d).

##### Blood transfusion rate

**Figure 4:**
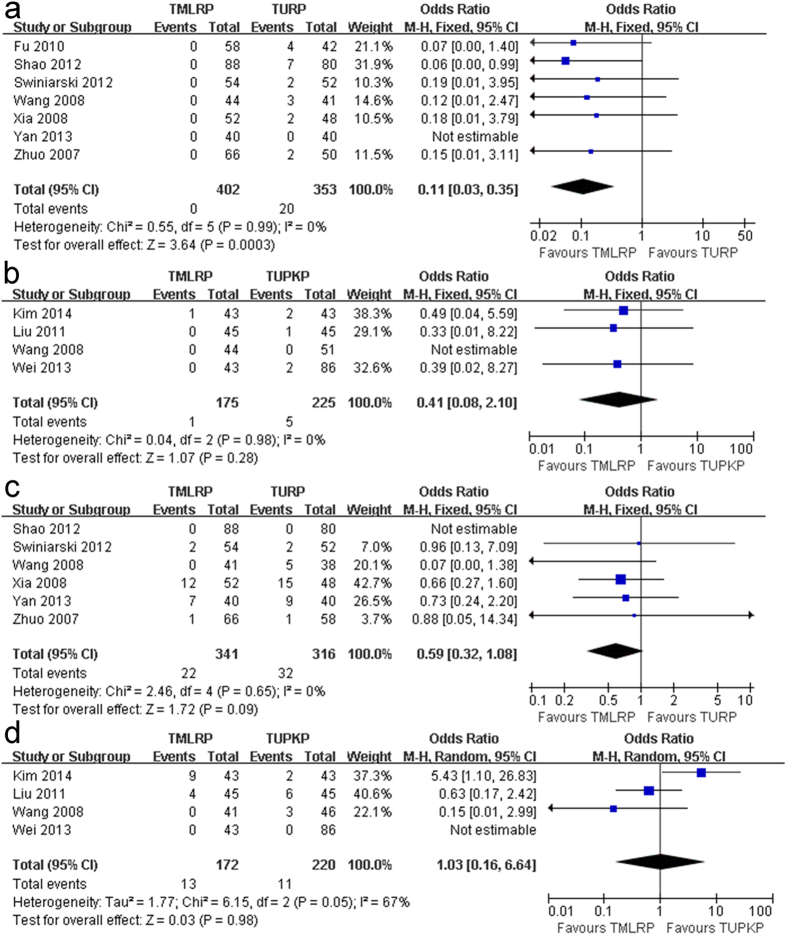
(**a**) Pooled estimate of blood transfusion between TMLRP and TURP. (**b**) Pooled estimate of blood transfusion between TMLRP and TUPKP. (**c**) Pooled estimate of transitory urge incontinence between TMLRP and TURP. (**d**) Pooled estimate of transitory urge incontinence between TMLRP and TUPKP.

The blood transfusion rate was recorded in 10 studies^11^,^13^,^15^,^16^,^17^,^19^,^20^,^21^,^22^,^24^ that included 1155 patients. Our meta-analysis indicated that the TMLRP group had a lower blood transfusion rate than the TURP group (odds ratio [OR]: 0.11; 95% CI: 0.03 to 0.35; *p* < 0.001), although no statistical heterogeneity was observed in this pooled analysis (I^2^: 0%; Fig. 4a). However, the pooled estimate showed no significant difference between the TMLRP group and the TUPKP group in terms of the blood transfusion rate (OR: 0.41; 95% CI: 0.08 to 2.10; *p *= 0.28; I^2^: 0%; Fig. 4b).

##### Local complication rate

**Figure 5:**
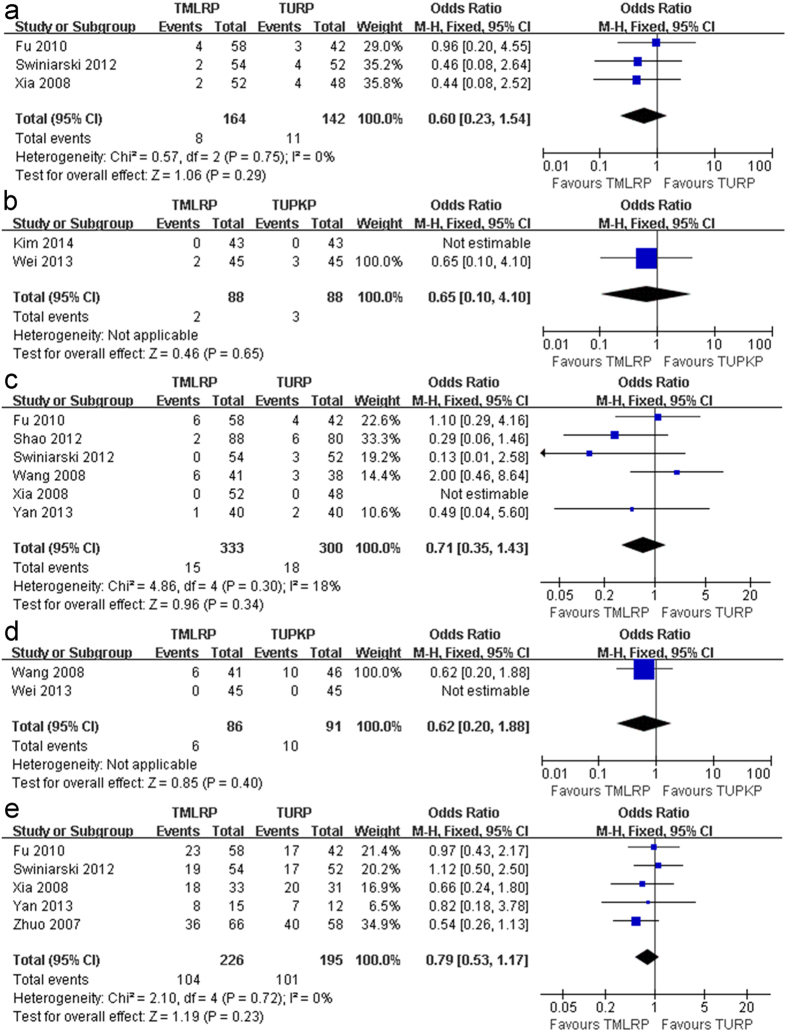
(**a**) Pooled estimate of UTI between TMLRP and TURP. (**b**) Pooled estimate of UTI between TMLRP and TUPKP. (**c**) Pooled estimate of recatheterization rate between TMLRP and TURP. (**d**) Pooled estimate of recatheterization rate between TMLRP and TUPKP. (**e**) Pooled estimate of retrograde ejaculation rate between TMLRP and TURP.

**Figure 6:**
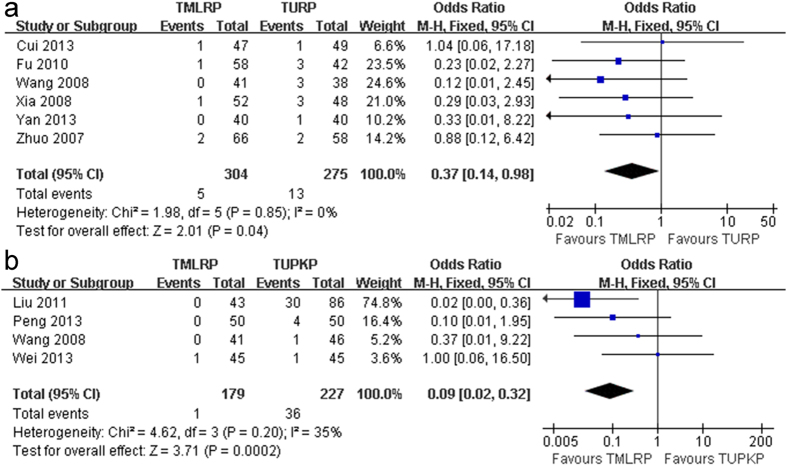
(**a**) Pooled estimate of urethral stricture rate between TMLRP and TURP. (**b**) Pooled estimate of urethral stricture rate between TMLRP and TUPKP.

There was no significant difference between TMLRP and either TURP or TUPKP in the transitory urge incontinence rate (OR: 0.59; 95% CI: 0.32 to 1.08; *p* = 0.09; I^2^: 0%; Fig. 4c and OR: 1.03; 95% CI: 0.16 to 6.64; *p* = 0.98; I^2^: 67%; Fig. 4d, respectively), urinary tract infection (UTI) rate (OR: 0.60; 95% CI: 0.23 to 1.54; *p* = 0.29; I^2^: 0%; Fig. 5a and OR: 0.65; 95% CI: 0.10 to 4.10; *p* = 0.65; Fig. 5b, respectively), and recatheterization rate (OR: 0.71; 95% CI: 0.35 to 1.43; *p *= 0.34; I^2^: 18%; Fig. 5c and OR: 0.62; 95% CI: 0.20 to 1.88; *p *= 0.40; Fig. 5d, respectively). In addition, there was no significant difference in the retrograde ejaculation rate (OR: 0.79; 95% CI: 0.53 to 1.17; *p* = 0.23; I^2^: 0%; Fig. 5e) between the TMLRP and TURP groups. However, the pooled estimates were significantly different between the TMLRP group and either the TURP or TUPKP group in terms of the urethral stricture rate (OR: 0.37; 95% CI: 0.14 to 0.98; *p* = 0.04; I^2^: 0%; Fig. 6a and OR: 0.09; 95% CI: 0.02 to 0.32; *p* < 0.001; I^2^: 35%; Fig. 6b, respectively).

## Discussion

To our knowledge, the present study is the first meta-analysis to compare the safety and efficacy of the TMLRP technique with either the TURP or TUPKP technique in patients with BPH. TURP is reportedly associated with a significant complication rate of 11.1%^2^. Moreover, TUPKP is a bipolar electrosurgical procedure that can notably reduce complications such as blood loss and other disadvantages associated with TURP^25^,^26^,^27^. Various laser treatment options have been developed in recent years. Recently, the thulium laser—a new type of surgical laser—is being increasingly applied in the urology field and overcomes many of the limitations of TURP and TUPKP, with encouraging efficacy and safety^28^,^29^. These new laser-based treatments can markedly improve the safety of patients and yield excellent results^28^.

Our meta-analysis showed that the TMLRP group exhibited ideal results as compared to the TUPKP and TURP group in terms of Qmax, PVR, IPSS, and QoL. And the QoL was slightly higher in the TMLRP group as compared to the TURP and TUPKP groups at the 1-month follow-up, and a significant difference was noted. All the micturition parameters of the three groups were similar at the subsequent follow-ups. Our study demonstrated that TMLRP was as effective as TURP and TUPKP in improving patient symptoms and urodynamic measurements postoperatively.

Compared with TURP or TUPKP, our meta-analysis demonstrated that TMLRP had a longer operation time. Xia *et al.*^22^ showed that TMLRP had a shorter operation time than TURP, although the difference was not significant. There may be three potential explanations for the longer operation time in the TMLRP group. First, the surgeons may have been more experienced in performing TURP or TUPKP techniques. In contrast, TMLRP is a newer technique, and although the procedure is easy to learn, surgeons need time to overcome the learning curve. Second, the resection volume of prostate tissue may be greater in the TMLRP group than in either the TURP or TUPKP group. Third, TMLRP combines the resection and simultaneous vaporization processes, thus resulting in a longer operation time for tissue-cutting^15^,^19^,^22^. Even though TMLRP had a longer operation time, our meta-analysis indicated that this technique was associated with a lower decrease in serum hemoglobin level, a shorter catheterization time and length of hospital stay, and a lower risk of local complications compared with the other two methods. In addition, our meta-analysis also demonstrated that TMLRP was associated with a lower blood transfusion rate than TURP.

The decrease in serum hemoglobin levels and blood transfusion rate was lower in the TMLRP group, as compared with the TURP or TUPKP group. In fact, among the TMLRP patients included in our meta-analysis, only one patient needed blood transfusion in the study by Kim *et al.*^11^. This finding may be explained by the excellent coagulation offered by these techniques, considering that the thulium laser wavelength is superior for controlling bleeding during the operation. The central wavelength of the thulium laser used in TMLRP can be adjusted between 1.75 and 2.22 μm, which enables the matching of this wavelength with the water absorption peak (1.92 μm) in tissue. The high density of absorbed energy at the tissue surface leads to instant vaporization and limits the penetration depth from 0.5 to 2 mm, thus indicating that the thulium laser may yield a sufficient hemostasis effect with minimal risk of thermal injury to surrounding tissue^29^. Due to sufficient hemostatic capacity, the thulium laser also provides a surprisingly visual field, along with low blood loss during surgery^30^. As an added benefit, the patients’ urine becomes clear more quickly after surgery, thus decreasing both the catheterization time and length of hospital stay. Of course, this may also be attributed to the decreased thermal damage and reduced scar formation as well as lower frequency of urethral stricture after the laser incision^31^.

We also performed a meta-analysis of the local complication rates between TMLRP and either TURP or TUPKP, including complications such as transitory urge incontinence, UTI, and recatheterization. The present meta-analysis indicated that there was a slightly better improvement of the abovementioned adverse events in the TMLRP group as compared with either the TURP or TUPKP group. In addition, the retrograde ejaculation rate was evaluated between the TMLRP and TURP groups, but no significant difference was noted. Although the findings of the present study support the results of previous clinical trials^11^,^12^,^13^,^14^,^15^,^16^,^17^,^18^,^19^,^20^,^21^,^22^,^23^,^24^,^32^, they still require verification in a large study.

Our meta-analysis does have certain limitations. Some of the results (such as Qmax, PVR, QoL, IPSS, operation time, decrease in hemoglobin level, length of hospital stay, and catheterization time) were associated with certain heterogeneities. These heterogeneities may be a result of several factors such as differences in prostate volume, operator skill, and follow-up duration; insufficient or unclear allocation concealment and blinding. In addition, a major limitation of this study may be our consideration of both thulium laser enucleation and thulium laser vaporesection as TMLRP. Another limitation was the limited number of well-constructed prospective trials; only four CCTs were included in our analysis. Furthermore, the difference in the resected weight of the prostate may have an influence on the results, particularly the operation time values. Nevertheless, we applied a sensitivity analysis to explore the reliability of our meta-analysis results. The results of this analysis did not indicate any substantial change in our initial conclusions. Thus, it also strengthened our level of confidence in the meta-analysis findings and credibility of the pooled results.

In conclusion, although our analysis found that TMLRP was associated with a longer operation time than either TURP or TUPKP, patients undergoing TMLRP might yield other benefits such as lower decreases in serum hemoglobin levels, shorter length of hospital stay and catheterization time, and a lower rate of urethral stricture. In addition, our analysis found that TMLRP was also associated with a lower blood transfusion rate than TURP. Moreover, TMLRP demonstrated similar efficacy in terms of Qmax, IPSS, PVR, and QoL at 1, 3, 6, and 12 months of postoperative follow-up and similar safety in terms of local complications such as transitory urge incontinence, UTI, and recatheterization as compared with either TURP or TUPKP. Our data suggest that TMLRP is a promising, minimally invasive technique that is a safe and feasible alternative to TURP or TUPKP for patients with BPH. Of course, more rigorously designed, larger, high-quality RCTs are required for further verification of these findings.

## Methods

### Search strategy

The Medline, EMBASE, Web of Science, and Google Scholar databases were independently searched by two reviewers in April 2014. This search used the following terms: thulium laser, TMLRP; transurethral plasmakinetic, TUPKP; transurethral resection of prostate, TURP; and benign prostatic hyperplasia, BPH. Our literature search had neither publication status nor language restrictions. In addition, the reviewing of each relevant article was independently performed by 2 reviewers.

### Inclusion and exclusion criteria

Relevant studies were included in this systematic review and meta-analysis if they met the following criteria: (1) compared TMLRP with either TUPKP or TURP, (2) clearly documented the indications for resection of the prostate, (3) provided data for at least one of the predefined outcome measurements. In contrast, studies were excluded if (1) the inclusion criteria were not met or (2) data were not provided or were impossible to calculate for TMLRP or TUPKP or TURP. All study titles and abstracts were independently screened by the same reviewers, and the complete texts were reviewed when deemed necessary. Discrepancies were resolved through consultation with another author.

### Data extraction

The following variables from each study were recorded independently by two reviewers: first author name, publication year, research design type, intervention method, total number of patients enrolled, patient age, prostate volume, PSA level, Qmax, PVR, QoL, IPSS, and follow-up period duration. In addition, the following outcome measures were extracted: operative time, hemoglobin level decrease, length of hospital stay, catheterization time, blood transfusion rate, and rates of local complications, with the latter including transitory urge incontinence, UTI, recatheterization, retrograde ejaculation, and urethral stricture. Discrepancies were resolved by reaching a consensus between all authors contributing to this review.

### Quality assessment

The quality of the RCTs included in this systematic review was assessed independently by two reviewers by using the Jadad scale score^33^, which ranges from 0 to 5 points—the higher the score, the better the quality indication. A study with a Jadad score of 3 points or more was considered as a high quality study. The Jadad score evaluates studies based upon their randomization, blinding, and descriptions of participant withdrawals and dropouts. CCTs that were included in the review were assessed through a modification of the Newcastle-Ottawa Scale^34^. The review scores ranged from 0 to 9 points for each trial; scores between 0 and 4 implied low-quality, while those between 5 and 9 implied high-quality. Discrepancies were resolved by consultation with another author.

### Statistical analysis

This meta-analysis was conducted using the Review Manager 5.2.0 (Cochrane Collaboration, Oxford, UK). For continuous data, the data were expressed as the MD with a 95% CI. For dichotomous data, the data were expressed as the OR with a 95% CI. In both cases, a *p* value of <0.05 was considered statistically significant. Heterogeneity was analyzed using a chi-square test with N-1 degrees of freedom, wherein an alpha value of 0.01 was used to imply statistical significance, in conjunction with the I^2^ test. When I^2^ was <50%, heterogeneity was deemed acceptable. In addition, a fixed-effects model was used for the meta-analysis; otherwise, a random-effect model was used.

## Additional Information

**How to cite this article**: DeCao, H. *et al.* Comparison between thulium laser resection of prostate and transurethral plasmakinetic resection of prostate or transurethral resection of prostate. *Sci. Rep.* **5**, 14542; doi: 10.1038/srep14542 (2015).

## Figures and Tables

**Table 1:** Basic features and quality assessments of the included studies.

Studies	Design	Intervention(I/C)	No. ofPatients	Age (years)	ProstateVolume (cc)	PSA(ng/ml)	Qmax (ml/s)	PVR (ml)	QoL	IPSS	Follow-up(months)	QualityScore
Kim,2014^11^	CCT	TMLRP/TUPKP	43/43	71.0 ± 7.1/70.5 ± 8.2	NA/NA	5.2 ± 4.0/4.6 ± 5.7	7.8 ± 4.3/8.4 ± 4.4	106.7 ± 114.5/92.4 ± 82.7	NA/NA	26.4 ± 5.7/25.0 ± 4.0	1	5
Yang,2013^12^	RCT	TMLRP/TUPKP	79/79	62.4 ± 7.2/61.4 ± 6.9	72.4 ± 21.2/69.2 ± 23.1	2.4 ± 1.2/2.3 ± 1.2	8.7 ± 2.8/9.1 ± 3.2	79.5 ± 29.3/72.4 ± 28.1	3.9 ± 1.2/4.9 ± 1.3	22.7 ± 4.3/23.4 ± 3.7	1,3,6,12,18	3
Wei,2013^13^	RCT	TMLRP/TUPKP	45/45	69.8 ± 8.1/69.0 ± 7.0	112.8 ± 28.3/115.0 ± 39.4	6.3 ± 3.9/5.3 ± 4.2	8.1 ± 3.2/7.9 ± 2.9	90.0 ± 50.4/96.8 ± 42.9	4.4 ± 0.8/4.5 ± 0.9	21.6 ± 6.7/21.1 ± 7.0	1,6,12,18	3
Peng,2013^14^	RCT	TMLRP/TUPKP	50/50	75.3 ± 8.1/74.6 ± 7.9	57.8 ± 11.9/58.2 ± 14.7	4.2 ± 3.1/3.8 ± 3.3	7.9 ± 4.3/8.2 ± 3.9	97.1 ± 34.5/88.0 ± 37.6	4.6 ± 1.2/4.5 ± 1.3	20.3 ± 7.8/19.3 ± 8.2	3	3
Liu,2011^15^	CCT	TMLRP/TUPKP	43/86	74.8 ± 7.6/73.2 ± 6.9	72.0 ± 18.8/68.0 ± 16.9	3.7 ± 1.3/3.4 ± 1.6	4.5 ± 1.8/3.6 ± 2.1	132.0 ± 42.0/148.0 ± 49.0	5.1 ± 0.8/5.8 ± 0.9	23.3 ± 4.7/27.2 ± 4.8	6	5
Wang,2008^16^	CCT	TMLRP/TURP/TUPKP	44/42/51	71.0(49–86)/71.0(49–86)/71.0(49–86)	NA/NA/NA	NA/NA/NA	6.5 ± 3.1/6.5 ± 2.8/6.8 ± 2.9	145.0 ± 50.4/125.0 ± 35.1/110.2 ± 40.2	4.6 ± 1.3/4.9 ± 1.1/4.7 ± 1.1	28.3 ± 2.3/27.9 ± 2.5/27.7 ± 2.4	3	5
Yan,2013^17^	RCT	TMLRP/TURP	40/40	72.5 ± 7.9/74.5 ± 6.5	52.9 ± 12.3/54.3 ± 11.1	2.6 ± 2.1/2.8 ± 2.1	7.5 ± 2.6/7.8 ± 2.8	73.8 ± 35.0/74.9 ± 35.6	NA/NA	21.7 ± 4.2/22.6 ± 5.6	3	3
Cui,2013^18^	RCT	TMLRP/TURP	47/49	67.8 ± 10.1/70.4 ± 7.0	48.0 ± 18.3/54.8 ± 27.4	3.4 ± 2.6/3.7 ± 2.7	8.6 ± 3.9/8.4 ± 3.4	91.9 ± 119.3/59.8 ± 106.4	4.4 ± 1.1/4.4 ± 1.0	21.1 ± 6.2/20.2 ± 6.7	12,24,36,48	3
Swiniarski,2012^19^	RCT	TMLRP/TURP	54/52	68.3 ± 6.8/69.3 ± 7.2	62.0 ± 23.7/66.5 ± 22.0	3.3 ± 2.0/3.7 ± 2.7	7.7 ± 3.5/8.5 ± 3.6	166.2 ± 110.5/152.0 ± 112.2	4.7 ± 1.0/4.9 ± 1.0	20.3 ± 2.5/20.8 ± 6.0	1,3	3
Shao,2012^20^	RCT	TMLRP/TURP	88/80	72.3 ± 6.3/71.1 ± 8.1	61.3 ± 16.8/59.6 ± 14.2	NA/NA	6.5 ± 2.1/6.8 ± 1.8	145.4 ± 98.4/137.5 ± 77.1	4.6 ± 1.5/1.8 ± 0.9	18.4 ± 6.2/19.2 ± 5.7	3,6,12	4
Fu,2010^21^	RCT	TMLRP/TURP	58/42	68.2 ± 8.9/65.8 ± 8.4	49.8 ± 10.4/48.2 ± 7.6	2.2 ± 1.4/2.4 ± 1.5	6.5 ± 1.8/7.3 ± 2.4	197.4 ± 23.6/186.8 ± 37.2	4.8 ± 0.6/4.4 ± 0.7	22.6 ± 4.5/21.2 ± 3.7	1,3,6,12	3
Xia,2008^22^	RCT	TMLRP/TURP	52/48	68.9 ± 7.7/69.3 ± 7.3	59.2 ± 17.7/55.1 ± 16.3	2.1 ± 1.1/2.3 ± 1.4	8.0 ± 2.8/8.3 ± 3.0	93.1 ± 32.1/85.0 ± 36.7	4.7 ± 0.9/4.5 ± 1.1	21.9 ± 6.7/20.8 ± 5.8	1,6,12	4
Jia,2009^23^	CCT	TMLRP/TURP	30/30	74.0 ± 5.5/72.7 ± 7.7	58.0 ± 7.5/54.0 ± 6.5	NA/NA	8.0 ± 4.0/7.5 ± 3.9	NA/NA	4.3 ± 0.8/4.2 ± 0.7	19.0 ± 8.3/20.1 ± 7.3	1	5
Zhuo,2007^24^	RCT	TMLRP/TURP	66/58	74.3 ± 7.2/73.7 ± 8.0	58.4 ± 12.5/56.6±14.1	3.2 ± 3.0/3.5 ± 3.4	7.8 ± 4.1/8.1 ± 4.4	93.1 ± 32.1/85.0 ± 36.7	4.5 ± 1.1/4.4 ± 1.3	19.1 ± 8.5/18.2 ± 9.2	3	3

RCT = randomized controlled trial; CCT = clinical controlled trial; TMLRP = thulium laser resection of the prostate; TUPKP = transurethral plasmakinetic prostatectomy; TURP = transurethral resection of the prostate; PSA = prostate-specific antigen; I/C = intervention group/control group; Qmax = maximum urinary flow rate; PVR = post-voiding residual urine volume; QoL = quality of life score; IPSS = international Prostate Symptom Score.

**Table 2:** Comparison of effectiveness between TMLRP and TUPKP or TURP.

Outcomes	TMLRP/TURP, TMLRP/TUPKP							
	No. ofstudies	No. of patients	P value	WMD (95% CI)	Heterogeneity			
					Chi^2^	df	P	I^2^(%)
Qmax (ml/s)								
1 month	4, 3	194/172, 167/167	0.15, 0.33	0.64[−0.22,1.51], 0.51[−0.50,1.52]	3.00, 3.88	3, 2	0.39, 0.14	0, 48
3 months	6, 3	348/311, 173/180	0.15, 0.06	0.39[−0.15,0.93], −0.77[−1.59,0.04]	5.98, 0.13	5, 2	0.31, 0.94	16, 0
6 months	3, 3	195/167, 167/210	0.65, 0.06	−0.32[−1.71,1.07], 0.86[−0.05,1.77]	4.71, 1.03	2, 2	0.09, 0.60	58, 0
12 months	4, 2	237/212, 123/124	0.70, 0.72	0.21[−0.87, 1.28], −0.34[−2.23,1.55]	0.32, 0.04	2, 1	0.85, 0.84	0, 0
PVR (ml)								
1 month	3, 3	164/142, 167/167	0.18, 0.66	2.01[−0.90,4.92], −0.73[−4.03,2.56]	7.23, 0.84	2, 2	0.03, 0.66	72, 0
3 months	5, 3	308/271, 173/180	0.41, 0.32	0.62[−0.84,2.07], −0.80[−2.38,0.79]	2.39, 2.08	4, 2	0.66, 0.35	0, 4
6 months	3, 3	195/167, 167/210	0.18, 0.05	−1.11[−2.76,0.53], 1.30[−0.01,2.62]	2.48, 0,15	2, 2	0.29, 0.93	19, 0
12 months	4, 2	237/212, 123/124	0.24, 0.55	−1.11[−2.94,0.73], −1.01[−4.32,2.31]	0.28, 0.01	2, 1	0.78, 0.93	0, 0
QoL								
1 months	4, 2	194/172, 124/124	<0.0001, 0.003	0.28[0.15, 0.40], 0.09[0.03,0.15]	1.03, 2.43	3, 1	0.79, 0.12	0, 59
3 months	5, 3	308/271, 173/180	0.60, 0.50	0.04[−0.12,0.21], −0.08[−0.30,0.14]	3.12, 6.12	3, 2	0.37, 0.05	4, 67
6 months	3, 3	195/167, 167/124	0.32, 0.88	0.20[−0.21,0.61], −0.01[−0.17,0.14]	0.00, 0.11	1, 1	1.00, 0.74	0, 0
12 months	4, 2	237/212, 123/124	0.74, 0.65	−0.04[−0.25,0.18], 0.05[−0.17,0.26]	13.02, 0.58	2, 1	0.001, 0.44	85. 0
IPSS								
1 months	4, 3	194/172, 167/167	0.54, 0.29	0.51[−1.14,2.17], −0.30[−0.85,0.25]	18.45, 2.56	3, 2	0.0004, 0.28	84, 22
3 months	6, 3	348/311, 173/180	0.20, 0.07	−0.23[−0.59,0.12], 0.31[−0.02,0.64]	4.41, 1.92	5, 2	0.49, 0.38	0, 0
6 months	3, 3	135/167, 167/210	0.79, 0.26	0.60[−0.35,0.46], 0.44[−0.33,1.20]	0.41, 1.05	2,1	0.81, 0.31	0, 5
12 months	4, 2	237/212, 123/124	0.32, 0.48	−0.28[−0.84,0.28], 0.43[−0.76,1.62]	0.22, 0.06	2, 1	0.90, 0.80	0, 0
